# Treatment Strategy for Chronic Obstructive Parotitis Related to Diabetes: A Retrospective Analysis of 12 Cases

**DOI:** 10.3389/fphar.2022.869872

**Published:** 2022-06-29

**Authors:** Chuan-Bin Wu, Lei Xue, Qing Zhou

**Affiliations:** Liaoning Provincial Key Laboratory of Oral Diseases, Department of Oral and Maxillofacial Surgery, School and Hospital of Stomatology, China Medical University, Shenyang, China

**Keywords:** diabetes mellitus, salivary glands, sialadenitis, Stensen’s duct, sialendoscopy

## Abstract

**Objectives:** The aim of this study was to describe our experience in treating chronic obstructive parotitis (COP) related to diabetes.

**Methods:** Twelve patients with COP related to diabetes were selected for the study. A sialendoscope was introduced from the orifice to investigate the ductal wall and lumen. During the operation, chymotrypsin and gentamicin were injected. All patients were followed up for 6 months. Preoperative and postoperative visual analog scale (VAS) evaluations and salivary gland scintigraphy (SGS) examinations were applied to evaluate the therapeutic effect; differences were considered statistically significant at *p* < 0.05.

**Results:** A sialendoscope was successfully used under local anesthesia in all members of the cohort. As shown by the endoscope, mucus plugs were the most common feature. Some adhered tightly to the ductal wall. We also found ductal congestion in some cases. The postoperative VAS scores and SGS counts were both significantly lower than the preoperative values (*p* < 0.05).

**Conclusion:** Chymotrypsin administration during interventional sialendoscopy is significantly effective in the treatment of diabetes-related COP.

## Introduction

Chronic obstructive parotitis (COP) is a common clinical disease in which the affected parotid gland repeatedly becomes swollen and painful, especially when patients consume sour food or when they eat in general (Wu et al., 2015a; Guo et al., 2017). Diabetes is a metabolic disease that is characterized by hyperglycemia and can directly or indirectly harm the body. The presence of diabetes and its complications significantly reduces the quality of life of patients (Liu et al., 2018; Cai et al., 2019). Diabetes-related oral disease, including periodontal disease, is also the sixth major complication of diabetes (Löe, 1993). In addition to periodontal disease, diabetic patients are also at risk of many other oral diseases and abnormalities, such as dental caries, candidiasis, gingivitis, tooth loss, neurosensory disorders, salivary gland dysfunction, dry mouth, and taste disorders (Negrato and Tarzia, 2010; Takeuchi et al., 2015). Among these conditions, dry mouth caused by decreased saliva secretion is one of the most common oral problems in diabetic patients and severely affects the quality of life. Many oral changes in diabetes are secondary to dry mouth. There is an urgent need for a technical method that can be applied clinically to alleviate dry mouth in patients. This article summarizes the application of sialendoscopy with chymotrypsin in the treatment of diabetes-related parotitis at our hospital and describes our treatment experience.

## Materials and Methods

### Clinical Materials

In this study, we retrospectively reviewed twelve patients. All patients were diagnosed with COP related to diabetes, and they all underwent sialendoscopy under local anesthesia. These patients visited the Department of Oral and Maxillofacial Surgery, School of Stomatology, China Medical University, from November 2020 to June 2021. In total, seven males and five females were recruited into the cohort. These participants ranged from 41 to 72 years old, with an average age of 62.4 years. The course of the disease ranged from 3 to 18 months, with an average of 8.3 months. All patients had diabetes; the duration of disease ranged from 6 to 20 years, with an average of 9.5 years (Table 1). We used ultrasonography to exclude possible causes of calculi, and salivary gland scintigraphy (SGS) was performed to assess salivary function.

**TABLE 1:** Characteristics of patients.

Number	Gender	Age (year)	Diabetes Time (year)	Type of diabetes	Course of disease (month)	Affected side	Symptoms	VAS	SGS
									UR (‰) EF (%)
1	F	66	8	I	5	L	Swelling, pain	5	1.49 55.35
						R			1.43 54.36
2	M	63	7	II	4	L	Swelling, pain	7	1.33 59.12
						R			1.41 58.97
3	M	57	9	II	7	L	Swelling, pain	6	1.32 57.12
						R			1.42 56.87
4	F	41	8	II	3	L	Swelling, pain	4	1.40 57.11
						R			1.38 52.66
5	M	55	8	II	6	L	Swelling, pain	7	1.42 51.22
						R			1.35 50.66
6	F	70	9	II	9	L	Swelling, pain	6	1.51 54.33
						R			1.62 50.22
7	M	58	6	I	10	L	Pain	6	1.49 55.66
						R			1.48 53.69
8	M	62	7	II	8	L	Pain	5	1.49 55.26
						R			1.47 56.31
9	M	68	18	II	18	L	Dry mouth	7	1.07 41.55
						R			1.21 41.21
10	F	71	6	II	7	L	Swelling, pain	6	1.49 51.23
						R			1.48 51.66
11	F	72	20	II	17	L	Dry mouth	7	0.92 44.74
						R			0.87 35.13
12	M	66	8	II	6	L	Swelling, pain	6	1.32 49.98
						R			1.39 52.11

Abbreviations: M, male; F, female; L, left parotid gland; R, right parotid gland; UR, uptake ratio; EF, excretion fraction.

### Operative Process

**FIGURE 1:**
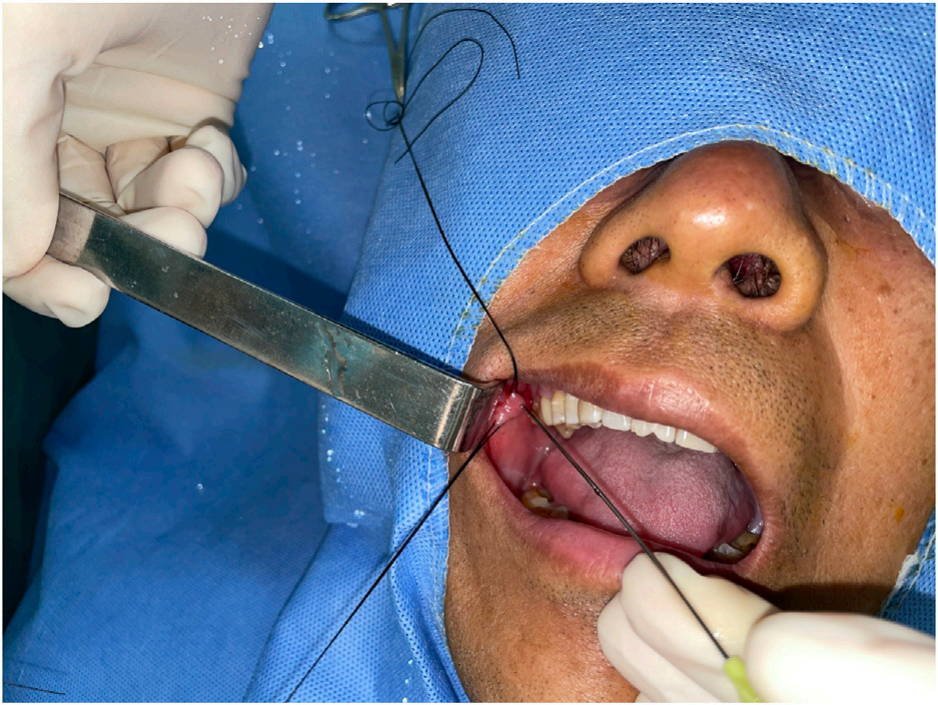
Surgical procedure: The sialendoscope was introduced through the orifice.

The surgeon injected the anesthetic around the orifice of the parotid canal on the affected side, and a set of blunt probes (sized 00 to 3; Polydiagnost, Germany) were introduced gradually from the orifice to dilate the ducts. Then, the sialendoscope was inserted (Figure 1). Guided by the image on the screen, the surgeon advanced the sialendoscope further into the canal. At the ductal bifurcating area, the sialendoscope was stopped. The chymotrypsin powder (10 mg) and gentamicin solution (100,000 units) were mixed with sterile saline solution (20 ml) and injected with a side-vented needle into the ductal system. The injection was performed fast, within 30 s. Then, the endoscope was pulled out; it was not introduced into the ductal system until 5 min later. At the end of the 5-min period, pressure was applied to the parotid region to expel the mucus plug from the orifice. Importantly, the pressure was applied before the reintroduction of the endoscope. The surgeon examined the image on the screen to ensure that the mucus plug was removed, especially if it had been tightly adhering to the wall previously. If the plug remained inside, the process was repeated. All patients were asked to return for additional flushing of the parotid canal after 1, 2, and 3 weeks.

### Assessment of Effectiveness Using the Visual Analog Scale

The VAS score was used to assess the effect of the method by evaluating clinical symptoms, including pain and swelling of the parotid gland, before and after treatment. The VAS ranges from 0 to 10, with a score of 0 indicating no clinical symptoms and a score of 10 indicating unbearable symptoms (Wewers and Lowe, 1990). All patients were evaluated using the VAS before surgery and 6 months after surgery. Also, the SGS was applied before and 6 months after surgery. A paired *t* test was conducted, and differences were considered statistically significant at *p* < 0.05.

### SGS Procedure

The patient was positioned supine with the chin raised. After intravenous injection of approximately 370 MBq (10 mCi) of 99mTc-pertechnetate, salivary gland scintigraphy was performed for 30 min with single photon emission computed tomography (e-cam 9550; Siemens Medical Solutions, Malvern, PA), using a low-energy, high-sensitivity, parallel hole collimator at 1 min per frame. Vitamin C, 0.2 g, was administered orally at 20 min to stimulate salivary gland excretion. The patient’s head was fixed during scintigraphy. Images were recorded in a 64 × 64 matrix. The energy window around the 140-keV photopeak of 99mTc was 20%.

## Results

All patients (twenty-four glands) successfully underwent interventional sialendoscopy under local anesthesia on the affected sides, and possible causes of calculi were excluded by ultrasonography examination. In the cohort, two had type I diabetes and the rest had type II diabetes. SGS showed that the uptake capabilities of the affected parotid glands were approximately normal, but the secretory function had decreased. After sialendoscopy, SGS revealed improved uptake and secretion in 10 patients (20 glands). The other two patients showed decreased uptake and secretion. The procedure duration varied from 18 to 26 min, with an average of 21.6 min. Mucus plugs were the most common feature identified by endoscopy. Swelling occurred in eight (66.7%) of the 12 patients; dry mouth was found in two patients (16.7%), and ten (83.3%) complained of pain. Compared with the preoperative score of 6, the mean VAS score 6 months after sialendoscopy was 4.3. The highest and lowest scores, respectively, were 7 and 4 preoperatively and 8 and 3 postoperatively. Twenty glands (83.3%) showed improved uptake and secretion on SGS.

**FIGURE 2:**
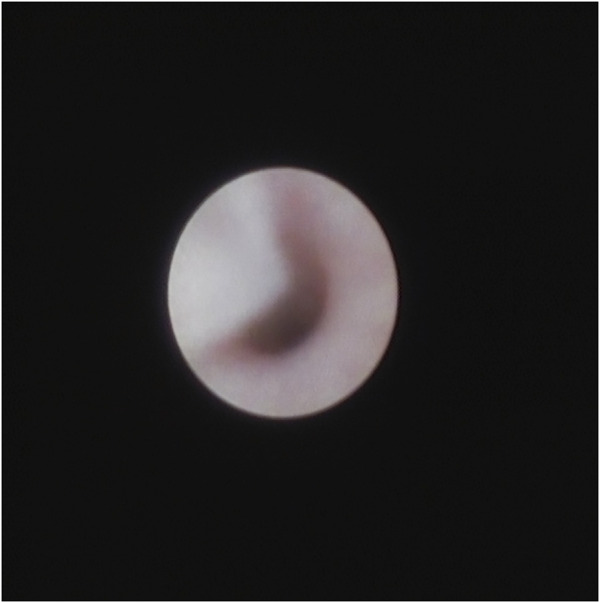
Sialendoscopic view: The duct was stenosis and the mucus plug was in the lumen.

Upon sialendoscopy, the wall of the canal was often observed to be congested, often having a mucosal plug, which contributed to the narrowing of the canal (Figure 2). In fact, some mucus plugs were so tightly adhered to the ductal wall that they could not be wiped off by a single back-and-forth movement of the endoscope.

The VAS scores of all except two patients were lower after the surgery than before; in one patient, the postoperative score was the same as the preoperative score, and in one patient, the postoperative score was higher than the preoperative score (*p* < 0.05). Additionally, the SGS counts of all except two patients were higher after the surgery than before (*p* < 0.05) (Table 2).

**TABLE 2:** Postoperative characteristics of patients.

Number	Affected side	Endoscopic findings	Procedure duration (minute)	Intervention	Complication	VAS	SGS UR (‰)EF (%)
1	L	MP	20	Removal + dilation + irrigation	None	3	1.62 61.01
	R						1.61 60.22
2	L	MP	18	Removal + dilation + irrigation	None	4	1.54 62.33
	R						1.58 64.21
3	L	MP	19	Removal + dilation + irrigation	None	3	1.45 68.12
	R						1.55 67.21
4	L	MP	22	Removal + dilation + irrigation	None	3	1.55 63.22
	R						1.48 64.55
5	L	MP	25	Removal + dilation + irrigation	None	5	1.51 65.21
	R						1.44 59.99
6	L	MP	23	Removal + dilation + irrigation	None	4	1.61 61.32
	R						1.70 59.98
7	L	MP	22	Removal + dilation + irrigation	None	4	1.55 62.55
	R						1.59 63.22
8	L	MP	19	Removal + dilation + irrigation	None	3	1.61 62.55
	R						1.59 63.12
9	L	Stenosis + MP	26	Removal + dilation + irrigation	None	8	0.99 40.11
	R						1.05 39.88
10	L	MP	24	Removal + dilation + irrigation	None	4	1.61 62.33
	R						1.62 61.22
11	L	Stenosis + MP	21	Removal + dilation + irrigation	None	7	0.88 40.32
	R						0.74 32.88
12	L	MP	20	Removal + dilation + irrigation	None	4	1.45 58.63
	R						1.52 61.21

Abbreviations: L, left parotid gland; R, right parotid gland; MP, mucus plug; UR, uptake ratio; EF, excretion fraction.

## Discussion

López-Pintor et al. found that the volume and flow rate of saliva were significantly lower in diabetic patients than in nondiabetic patients, and the incidence of xerostomia was much higher in diabetic patients (12.5–53.5%) than in nondiabetic people (0–30%) (López-Pintor et al., 2016). Lima et al. conducted a cross-sectional study on the oral health status of 120 patients with type 2 diabetes who had been diagnosed and treated for more than 1 year at the Diabetes Hypertension Center in Fortaleza, Brazil; 111 (92.5%) of them had obvious decrease in salivation, but as a subjective symptom, only 59 people (49.2%) reported obvious dry mouth (Lima et al., 2017).

COP affects the parotid canal system, with irregular expansion of the main canal and branches. The clinical symptoms include repeated swelling of the parotid, purulent or colloidal secretion from the opening of the canal, and aggravation of the symptoms by certain foods (Wu et al., 2015b; Abdullayev et al., 2016; Plonowska et al., 2019).

Many causes, such as sialolithiasis, ductal stricture, infection, or injury, could lead to chronic obstructive parotitis. A number of articles have reported the effectiveness of sialendoscope in the treatment of COP caused by routine factors (Wu et al., 2015b; Sun et al., 2017). COP related to diabetes is common in clinic but was rarely reported. In fact, there is no definition of parotitis related to diabetes. Our team believes that the specific nature of diabetes determines the specific nature of treatment for the disease. We therefore summarize our experience in the treatment of COP related to diabetes. The relevant scientific literature is relatively small. If this article is published, it will contribute to similar literature. According to our data, the most common feature of the disease on sialendoscopy is the presence of mucus plugs, some of which adhere to the wall of the lumen. It is difficult to remove tightly adhered mucus plugs using simple sialendoscopic dilation and irrigation. Thus, we introduced chymotrypsin. Chymotrypsin is a proteolytic agent that can solubilize floccules, mucus plugs, and shed epicytes. Normal saline with chymotrypsin and gentamicin can not only solubilize impurities in the canal but also diminish inflammation (Sun et al., 2017).

This article concludes with a description of our experience with sialendoscopic treatment at our hospital. The curative effect was poor in one patient and absent in another patient. Upon further investigation, we found that these two patients had suffered from diabetes longer than the other patients and that the uptake and secretory functions of the affected parotid were in mild or moderate decline. Patients with diabetes are prone to bacterial infections. In our view, the uptake function of the parotid can be damaged in patients with COP if treatment is not administered in a timely manner after the occlusion of the canal. The decline in parotid uptake function can lead to a decline in salivation, which contributes to a decline in flushing of the canal and aggravates occlusion of the canal, beginning a vicious cycle.

The patients were advised to undergo additional flushing of the parotid canal after 1, 2, and 3 weeks. This does not mean that they underwent additional sialendoscopic treatments. We flushed the canals with sterile saline solution as an outpatient treatment. We suspected that the surgical region might be swollen after endoscopic treatment, narrowing the ductal system. Thus, it would be beneficial for patients to receive additional ductal flushing. Our team encourages the SGS examination, which is noninvasive and accurate. The SGS helps doctors assess parotid function qualitatively and quantitatively. Again, we recommend the widespread use of SGS examination in the clinic (Markusse et al., 1993; Wu et al., 2015c; Kaldeway et al., 2019).

In summary, the study demonstrates that COP with diabetes can be effectively treated by interventional sialendoscopy with chymotrypsin and is very important to diagnose and treat early. However, this article has certain limitations. The number of cases is small and the follow-up time is short. We hope to increase the number of cases and extend the follow-up time in future work to further validate the efficacy of the technique.

## Data Availability

The original contributions presented in the study are included in the article/Supplementary Material; further inquiries can be directed to the corresponding author.
